# Author Correction: Neuronal Nsun2 deficiency produces tRNA epitranscriptomic alterations and proteomic shifts impacting synaptic signaling and behavior

**DOI:** 10.1038/s41467-021-27501-3

**Published:** 2021-12-08

**Authors:** J. Blaze, A. Navickas, H. L. Phillips, S. Heissel, A. Plaza-Jennings, S. Miglani, H. Asgharian, M. Foo, C. D. Katanski, C. P. Watkins, Z. T. Pennington, B. Javidfar, S. Espeso-Gil, B. Rostandy, H. Alwaseem, C. G. Hahn, H. Molina, D. J. Cai, T. Pan, W. D. Yao, H. Goodarzi, F. Haghighi, S. Akbarian

**Affiliations:** 1 Department of Neuroscience, Icahn School of Medicine at Mt. Sinai, New York, NY USA; 2 Friedman Brain Institute, Icahn School of Medicine at Mt. Sinai, New York, NY USA; 3 Department of Biochemistry and Biophysics, University of California San Francisco, San Francisco, CA USA; 4 Departments of Psychiatry and Behavioral Sciences, Neuroscience and Physiology, Upstate Medical University, Syracuse, NY USA; 5 The Rockefeller University Proteomics Resource Center, The Rockefeller University, New York, NY USA; 6 Department of Psychiatry, Icahn School of Medicine at Mt. Sinai, New York, NY USA; 7 Department of Biochemistry and Molecular Biology, University of Chicago, Chicago, IL USA; 8 Department of Neurosciences, Thomas Jefferson University, Philadelphia, PA USA; 9 Research and Development Service, James J. Peters Veterans Affairs Medical Center, Bronx, NY USA

**Keywords:** Depression, Epigenetics and behaviour, Epigenetics and plasticity, Molecular neuroscience

Correction to: *Nature Communications* 10.1038/s41467-021-24969-x, published online 13 August 2021.

The original version of this Article contained an error in the data availability statement, which incorrectly read. ‘The raw and processed mouse RNA sequencing data generated in this study have been deposited in the Gene Expression Omnibus database under accession code GSE165202’. The correct version adds, ‘tRNA, YAMAT, and Ribosome’ after ‘RNA’. This has been corrected in both the PDF and HTML versions of the Article.

The sequencing datasets deposited in GEO associated with this Article were updated shortly after publication to include the tRNA, YAMAT, and Ribosome sequencing data.

