# Supplementary material for: Neuronal Nsun2 deficiency produces tRNA epitranscriptomic alterations and proteomic shifts impacting synaptic signaling and behavior
Source: Nat Commun. 2021 Aug 13;12:4913. doi: 10.1038/s41467-021-24969-x (PMC8363735; doi:10.1038/s41467-021-24969-x)
Supplement: Supplementary file 1 — Supplementary Information [file 41467_2021_24969_MOESM1_ESM.pdf]

Supplementary Information

Blaze et al.

Neuronal Nsun2 deficiency produces tRNA epitranscriptomic alterations and proteomic shifts impacting synaptic signaling and behavior

Supplementary Figures

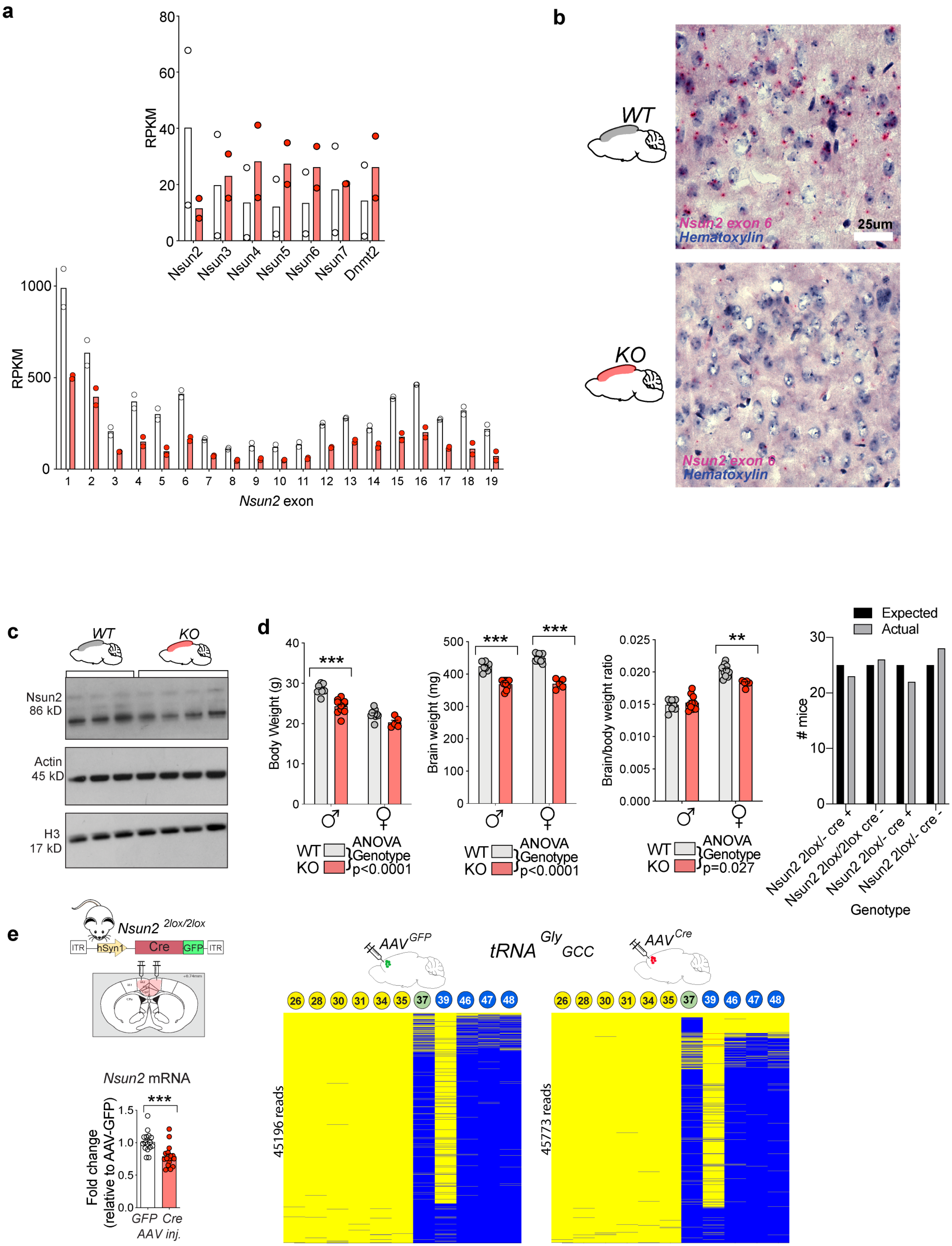
a
RPKM
Nsun2
Nsun3
Nsun4
Nsun5
Nsun6
Nsun7
Dnmt2
Nsun2 exon
b
WT
KO
Nsun2 exon 6
Hematoxylin
25um
c
Nsun2 86 kD
Actin 45 kD
H3 17 kD
d
Body Weight (g)
Brain weight (mg)
Brain/body weight ratio
WT
KO
ANOVA Genotype p<0.0001
ANOVA Genotype p<0.0001
ANOVA Genotype p=0.027
Expected
Actual
mice
Nsun2 2lox/- cre +
Nsun2 2lox/2lox cre -
Nsun2 2lox/- cre +
Nsun2 2lox/- cre -
Genotype
e
Nsun2 2lox/2lox
ITR
hSyn1
Cre
GFP
ITR
Nsun2 mRNA
Fold change (relative to AAV-GFP)
GFP
Cre
AAV inj.
AAV GFP
AAV Cre
tRNA Gly GCC
45196 reads
45773 reads

Supplementary Figure 1. (a) Top, RNA-seq RPKM for the family of *Nsun* genes and *Dnmt2* in cortex Nsun2 KO & WT mice. Bottom, RNA seq RPKM for individual *Nsun2* exons (n=2/genotype). **(b)** In situ hybridization of *Nsun2 exon 6* mRNA in WT (top) and Nsun2 KO (bottom) adult mouse cortex Layer II/III. *Nsun2 exon 6* mRNA is stained with pink and nuclei are stained blue with hematoxylin. Scale bar = 25 um. Experiment was replicated independently a second time in another pair of WT/KO mice with same results. **(c)** Uncropped immunoblots of WT and Nsun2 KO forebrain samples depicted in Fig. 1a. Experiment was replicated independently a second time in a different cohort of animals and same results were obtained. **(d)** Left, Body and brain weights for Nsun2 KO vs. WT mice [n=8 WT M, 10 WT F, 10 KO M, 5 KO F; two-tailed t-test with Bonferonni correction for post hoc comparisons of KO vs. WT (**p=0.005 and ***p<0.0001)]. Right, Mendelian ratios of observed vs. expected offspring genotypes (X^2(3, N=99) =0.459, p=0.928). Data are presented as mean values +/- SEM. **(e)** Top left, $Nsun2^{2lox/2lox}$ mice were injected to $AAVh^{Syn1\text{-}CreGFP}$ for PFC-specific conditional KO. Schematic representation of viral vector containing *hSyn1* promoter to drive neuronal expression, *Cre-recombinase*, and a *GFP* fusion protein (top). Brain atlas depiction of discrete PFC bilateral microinjections. Bottom left, A significant decrease in *Nsun2* mRNA was detected by qPCR in PFC tissue after $AAV8^{hSyn1\text{-}CreGFP}$ injection compared to $AAV8^{hSyn1\text{-}GFP}$ controls using *Gapdh* as a housekeeping gene (two-tailed t-test, ***p=0.0004; n=16 $AAV8^{hSyn1\text{-}GFP}$, 17 $AAV8^{hSyn1\text{-}CreGFP}$). Data are presented as mean values +/- SEM. Right, Representative methylation maps demonstrating decrease in $tRNA^{Gly}_{GCC}$ methylation following $AAV8^{hSyn1\text{-}CreGFP}$ injection (n=3 $AAV8^{hSyn1\text{-}GFP}$, 3 $AAV8^{hSyn1\text{-}CreGFP}$; individual cytosine analysis in Supplementary Table 6). Source data are available as a Source Data file.

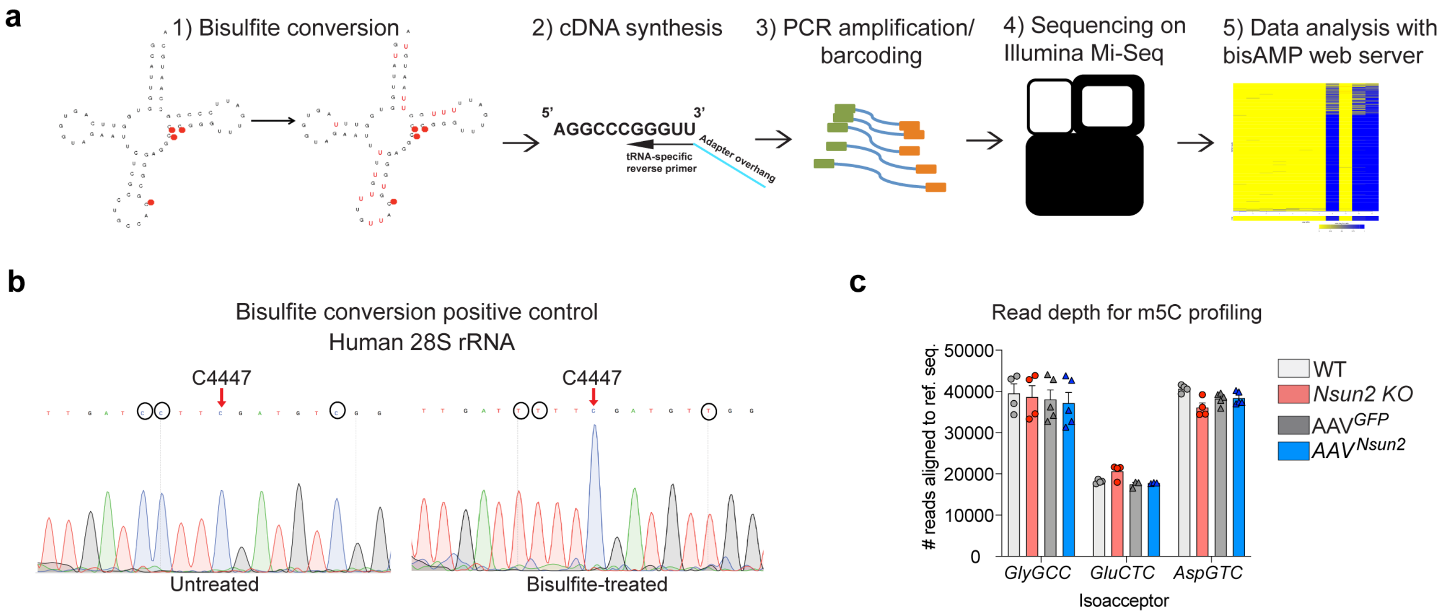

Supplementary Figure 2. (a) Method for targeted bisulfite sequencing of tRNAs to identify cytosine methylation levels using next-generation sequencing and the webserver bisAMP for data analysis. **(b)** Human 28S rRNA was used as a validated positive control for bisulfite conversion efficiency. Electropherograms from Sanger sequencing after RNA bisulfite conversion (right) or untreated RNA (left) show conversion of unmethylated cytosines to uracil (thymine) after bisulfite treatment while cytosine 4447 (C4447) remained unconverted after bisulfite treatment and confirmed ~100% methylation. **(c)** Total reads aligned to reference sequence after MiSeq run for each isoacceptor and condition. RNA from the same samples was used to run tRNA bisulfite sequencing for all 3 isoacceptors (n=4 WT, 4 KO for all isoacceptors; n=5 AAV-GFP, 5 AAV-Nsun2 for tRNA$^{Gly}{}_{GCC}$ and tRNA$^{Asp}{}_{GTC}$; n=3 AAV-GFP, 3 AAV-Nsun2 for tRNA$^{Glu}{}_{CTC}$). A two-way ANOVA revealed that while there was an effect of isoacceptor on aligned reads ($F(2,38)=169.3$, $p<0.0001$

0, there was no effect of genotype/AAV injection on reads aligned to the reference sequence ($F(3,38)=0.597$, $p=0.621$; Interaction $F(6,38)=0.8514$, $p=0.539$). Data are presented as mean values +/- SEM. Source data are available as a Source Data file.

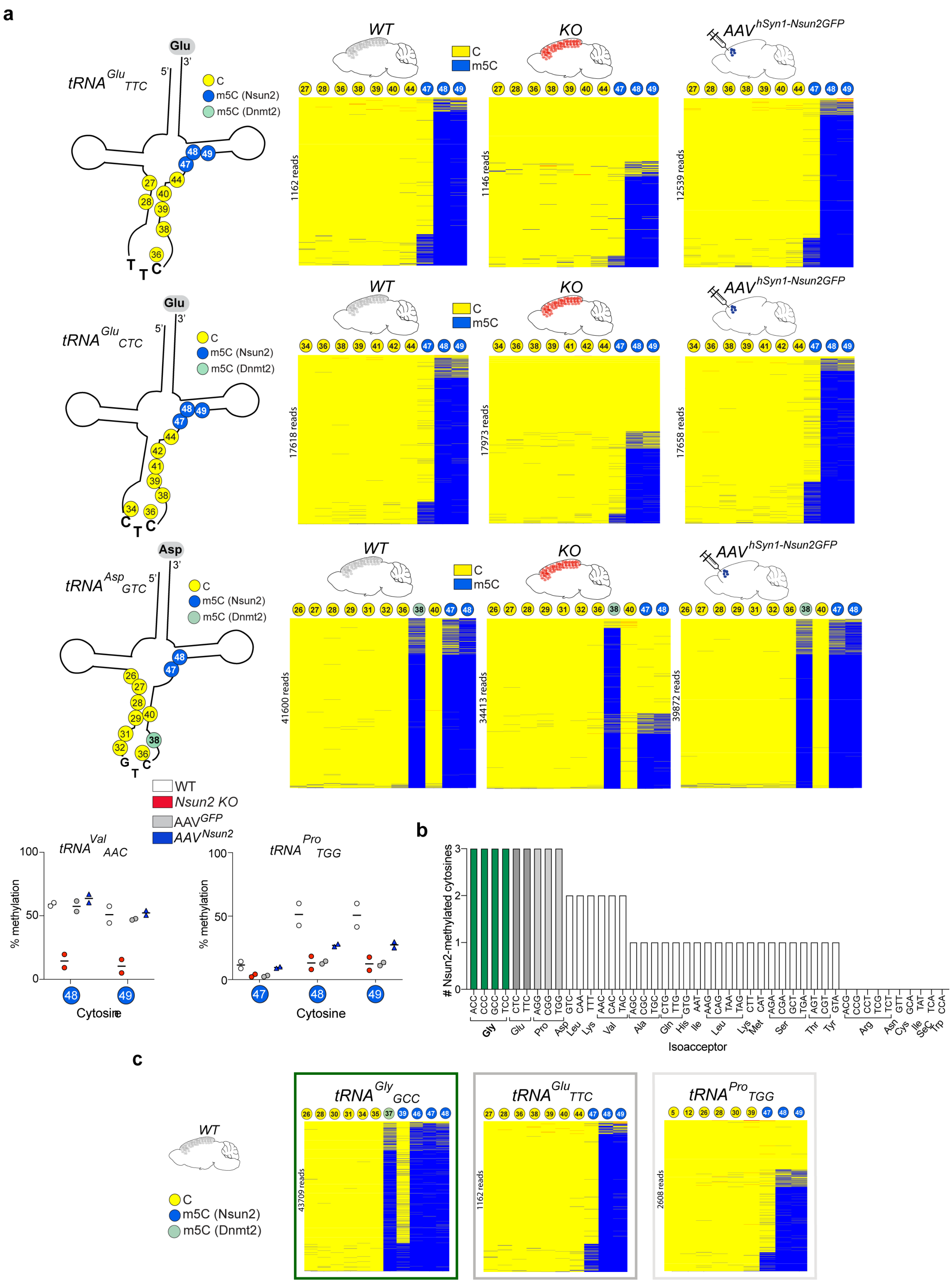
a
tRNA Glu TTC
tRNA Glu CTC
tRNA Asp GTC
C
m5C (Nsun2)
m5C (Dnmt2)
WT
KO
AAV hSyn1-Nsun2GFP
1162 reads
1146 reads
12539 reads
17618 reads
17973 reads
17658 reads
41600 reads
34413 reads
39872 reads
WT
Nsun2 KO
AAV GFP
AAV Nsun2
tRNA Val AAC
tRNA Pro TGG
% methylation
Cytosine
b
Nsun2-methylated cytosines
Isoacceptor
c
tRNA Gly GCC
tRNA Glu TTC
tRNA Pro TGG
43709 reads
1162 reads
2608 reads

Supplementary Figure 3. (a) Left, Schematics of $tRNA^{Glu}_{TTC}$, $tRNA^{Glu}_{CTC}$ and $tRNA^{Asp}_{GTC}$, depicting 10-11 cytosines that were queried with tRNA bisulfite sequencing. Top right, Representative individual methylation maps for WT, Nsun2 KO, and AAV^{Nsun2} as described in Figure 1. Bottom left, Nsun2-methylated sites for $tRNA^{Val}_{AAC}$ and $tRNA^{Pro}_{TTG}$ in n=2 mice/genotype or AAV injection. **(b)** Data from Blanco and colleagues[10] demonstrating number of Nsun2-methylated cytosines for each isoacceptor in mouse skin from bisulfite sequencing. **(c)** Methylation maps of our data from WT control mice for the three tRNA families with 3 or more Nsun2 methylation sites, including $tRNA^{Gly}_{GCC,}$ $tRNA^{Glu}_{TTC}$, and $tRNA^{Pro}_{TGG.}$ Source data are available as a Source Data file.

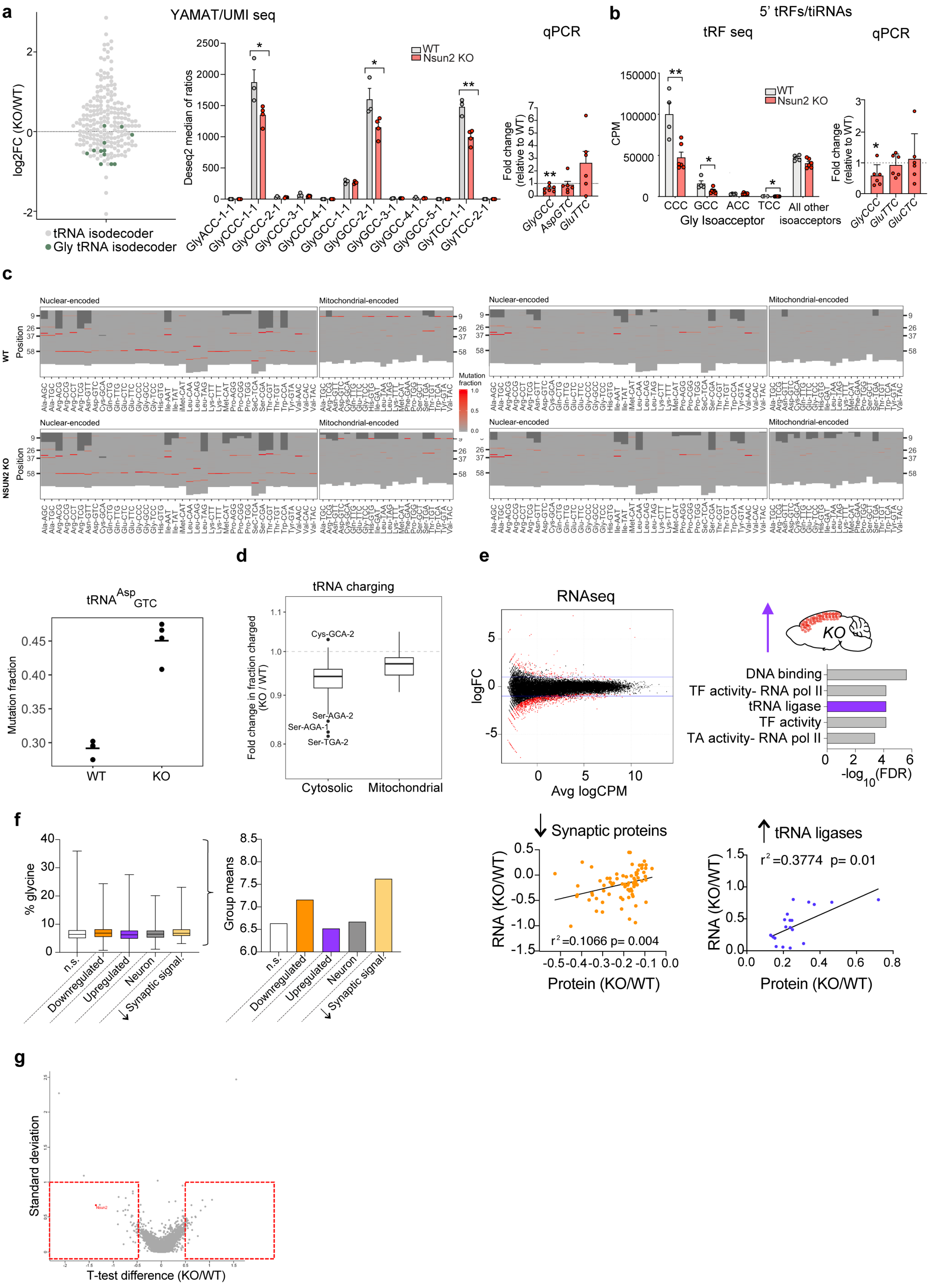
a
YAMAT/UMI seq
qPCR
log2FC (KO/WT)
tRNA isodecoder
Gly tRNA isodecoder
Deseq2 median of ratios
WT
Nsun2 KO
Fold change (relative to WT)
GlyGCC
AspGTC
GluTTC
b
5' tRFs/tiRNAs
tRF seq
qPCR
CPM
Gly Isoacceptor
All other isoacceptors
GlyCCC
GluTTC
GluCTC
c
Nuclear-encoded
Mitochondrial-encoded
WT
NSUN2 KO
Position
Mutation fraction
tRNA Asp GTC
Mutation fraction
WT
KO
d
tRNA charging
Fold change in fraction charged (KO / WT)
Cys-GCA-2
Ser-AGA-2
Ser-AGA-1
Ser-TGA-2
Cytosolic
Mitochondrial
e
RNAseq
logFC
Avg logCPM
KO
DNA binding
TF activity- RNA pol II
tRNA ligase
TF activity
TA activity- RNA pol II
-log10(FDR)
Synaptic proteins
tRNA ligases
RNA (KO/WT)
Protein (KO/WT)
r2 =0.1066 p= 0.004
r2 =0.3774 p= 0.01
f
% glycine
Group means
n.s.
Downregulated
Upregulated
Neuron
Synaptic signal.
g
Standard deviation
T-test difference (KO/WT)
Nsun2

Supplementary Figure 4. (a) Left, YAMAT/UMI seq results confirmed decreases in various glycine isodecoders in Nsun2 KO vs. WT (n=3 WT, 4 KO; two-tailed t-tests, Gly-CCC-1-1 *FDR adj. p=0.036, Gly-GCC-2-1 *FDR adj. p=0.041, Gly-TCC-1-1 **FDR adj. p=0.009). Right, qPCR (using *5S rRNA* as a housekeeping gene) further confirmed decreased $tRNA^{Gly}_{GCC}$ expression in KO vs. WT (n=6 WT, 7 KO; **p=0.008 vs. WT in one-sample t-test, two-tailed) while no changes between groups were detected for $tRNA^{Glu}_{TTC}$ (n=7 WT, 6 KO) and $tRNA^{Asp}_{GTC}$ (n=6 WT, 6 KO). Data are presented as mean values +/- SEM. **(b)** Left, tRNA fragment sequencing results from Nsun2 knockout and WT forebrain (n=4 WT, 6 KO). Sequencing reads aligned to 175 unique tRNA fragments originating from the 5' end, ranging from 14-34 nucleotides in length. This included fragments from 32 distinct tRNA isoacceptors coding for 15 different amino acids. We also identified 140 unique tRNA fragments originating from the 3' end only ranging from 17-22 nucleotides in length. The majority of reads were from 5' tiRNAs and tRFs originating from $tRNA^{Gly}$, and among the $tRNA^{Gly}$ isoacceptors, we detected a significant decrease in the mutant cortex for $tRNA^{Gly}_{CCC}$ (t(8)=3.808, **p=0.005), $tRNA^{Gly}_{GCC}$(t(8)=2.65, *p=0.029), and $tRNA^{Gly}_{TCC}$(t(8)=2.323, *p=0.049), but no change for $tRNA^{Gly}_{ACC}$(t(8)=0.767, p>0.999) or the sum of all other isoacceptors' fragments/halves (t(8)=2.10, p=0.069). Right, Custom small RNA assays were used for qPCR validation of tRF changes (n=7 WT, 6 KO for all isoacceptors), confirming the decrease of a highly expressed 5'tRF for $tRNA^{Gly}_{CCC}$ (two-tailed; t(5)=2.854, *p=0.036) while no changes were detected for two other highly expressed tiRNAs from $tRNA^{Glu}_{TTC}$(two-tailed; t(5)=0.5035, p=0.636) or $tRNA^{Gly}_{CTC}$ (two-tailed; t(5)=0.3736, p=0.724). Data are presented as mean values +/- SEM. **(c)** Other non-m^5C tRNA modifications. Top left, Heatmap shows mutation fractions at each tRNA position for the samples not treated with demethylase (n=4/genotype). Nuclear-encoded (cytosolic) and mitochondrial-encoded tRNAs are shown separately. For nuclear-encoded tRNAs only the most abundant isodecoder in each isoacceptor family is shown. Positions of coverage below the set threshold are shown in dark gray. The most common detected modifications are m^1A, m^3C and m^1G. Top right, Heatmap shows mutation fractions at each tRNA position for the same samples from left heatmap treated with demethylase. Demethylase removes the methyl group of $m^1A/m^3C/m^1G$, resulting in the elimination or reduction in mutation fractions, thus validating the presence of these Watson-Crick methylations. The remaining positions with high mutation fractions are known A-to-inosine modification sites. Bottom left, m^1A58 modification fraction of $tRNA^{Asp}$. m^1A mutation fraction can be used as a proxy for modification fractions[33]. Data are for the most

abundant $tRNA^{Asp}$ isodecoder. $tRNA^{Asp}$ is a known substrate for Nsun2, and an increase in m^1A58 modification in the KO mice may reflect partial compensation for the loss of m^5C modification in this tRNA. **(d)** tRNA charging by DM-tRNA-seq. Box and whisker plot shows the charging ratios of Nsun2 KO/WT mice (n=4/genotype) in nuclear-encoded or cytosolic tRNAs (median: 0.9419, IQR: 0.0429, whiskers represent +/- 1.5*IQR, ymin: 0.8594 and ymax=1.0099) and mitochondrial tRNAs (median: 0.9708, IQR: 0.0404, whiskers represent +/- 1.5*IQR, ymin: 0.9069 and ymax=1.0101). Data beyond the whiskers are outlying points. **(e)** RNA sequencing of Nsun2 KO cortex vs. WT (n=2/genotype) revealed 182 genes upregulated and 905 genes downregulated (red; FDR adj. p<0.05). Gene ontology analysis on StringDB identified tRNA aminoacyl ligase genes as one of the top 5 molecular function networks enriched in the upregulated genes after Nsun2 KO. Bottom, simple linear regression was used to calculate the relationship between subsets of enriched proteins from GO categories for upregulated tRNA ligase expression and downregulated synaptic protein expression from RNAseq data vs. mass spectrometry protein expression. **(f)** Left, Box plot with percent glycine amino acid content in proteins identified in our unbiased proteomics screen (see statistics for first three panels in box plot in Fig. 2c), including n=434 proteins with GO annotations denoting them as neuronal (median: 6.48, IQR: 2.485, whiskers ymin:1.117 and ymax:20.13). Final bar represents n=50 downregulated proteins known to be involved in excitatory synaptic signaling that may contribute to the electrophysiological phenotype observed in Nsun2 KO mice (median: 6.831, IQR: 2.283, whiskers represent absolute min and max, ymin: 3.107 and ymax: 23.08). Right, group means from left panel. **(g)** Mass spectrometry protein expression data from Fig. 2b with KO vs. WT differences plotted against standard deviations for each protein. The standard deviation was calculated by selecting three random, but non-repeating, replicates from each group and taking the average from them. Then a simple standard deviation was calculated between the two averages to provide a KO vs. WT ratio plotted against a standard deviation in a randomized matter. Proteins of interest all lie within the dashed red boxes with low standard deviations. Source data are available as a Source Data file.

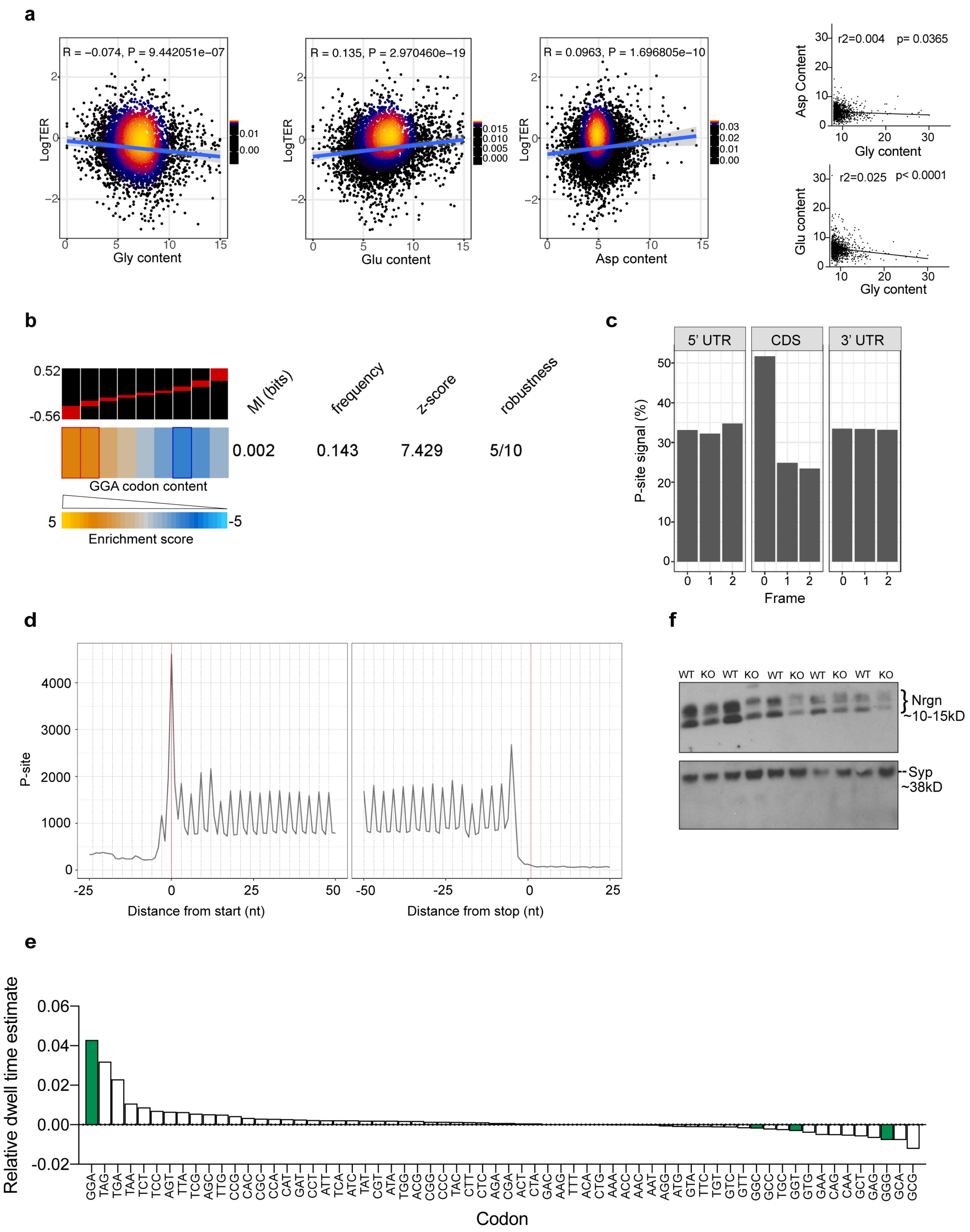
a
R = −0.074, P = 9.442051e−07
R = 0.135, P = 2.970460e−19
R = 0.0963, P = 1.696805e−10
LogTER
Gly content
Glu content
Asp content
r2=0.004 p= 0.0365
Asp Content
r2=0.025 p< 0.0001
Glu content
b
0.52
-0.56
MI (bits)
frequency
z-score
robustness
0.002
0.143
7.429
5/10
GGA codon content
5
-5
Enrichment score
c
5' UTR
CDS
3' UTR
P-site signal (%)
Frame
d
P-site
Distance from start (nt)
Distance from stop (nt)
f
WT KO WT KO WT KO WT KO WT KO
Nrgn
~10-15kD
Syp
~38kD
e
Relative dwell time estimate
Codon
GGA TAG TGA TAA TCT TCC AGT TTA TCG AGC TTG CCG CAC CGC CCA CAT GAT CCT ATT TCA ATC TAT CGT ATA TGG ACG CGG CCC TAC CTT CTC AGA CGA ACT CTA GAC AAG TTT ACA CTG AAA ACC AAC AAT AGG ATG GTA TTC TGT GTC GTT GGC GCC TGC GGT GTG GAA CAG CAA GCT GAG GGG GCA GCG

Supplementary Figure 5. (a) Left, LogTERs were plotted against glycine codon content for Nsun2 KO vs. WT forebrain and Pearson correlation was computed for glycine (including all Gly codons), which confirms glycine enrichment in less efficiently translated proteins (low logTERs; anti-correlation) from Fig. 2d. Asp and Glu codon content were also analyzed and did not show the same anti-correlation as Gly. However, Asp and Glu codon content did show a positive correlation with logTER. Right, in the top quartile of glycine-rich proteins, there is a negative correlation with Asp and Glu codon content, which may be why Asp and Glu show the opposite relationship to logTERs. **(b)** LogTER (generated from Riboseq data n=2 KO/2 WT) vs. GGA codon content (corresponding to TCC anticodon for Gly). Distribution of top 20% of GGA-enriched transcripts in our RiboSeq data (logTER Nsun2 KO vs WT) showing a significant enrichment of GGA-enriched transcripts among the low logTER mRNAs, and de-enrichment in the high logTER mRNAs. **(c,d)** Quality control data from Riboseq (Representative data from Nsun2 KO rep 2). **(c)** Percentage of P-sites in the 5' UTR, CDS and 3' UTR of mRNAs in three frames from Riboseq data, demonstrating most recovered fragments have their P-site in frame of the coding sequence and are distributed randomly in UTRs. **(d)** P-site signal for reads aligning near the start (left panel) and the stop (right panel) codons, confirming periodicity of the signal and typical accumulation on the start codon and on a penultimate codon. **(e)** Relative dwell time estimate for KO vs. WT cortex for each of the 64 codons. Note Gly codon GGA with the highest dwell time compared to all other codons. **(f)** Full-length immunoblots for Neurogranin and Synaptophysin in synaptosomes (Related to Fig. 4b). Source data are available as a Source Data file.

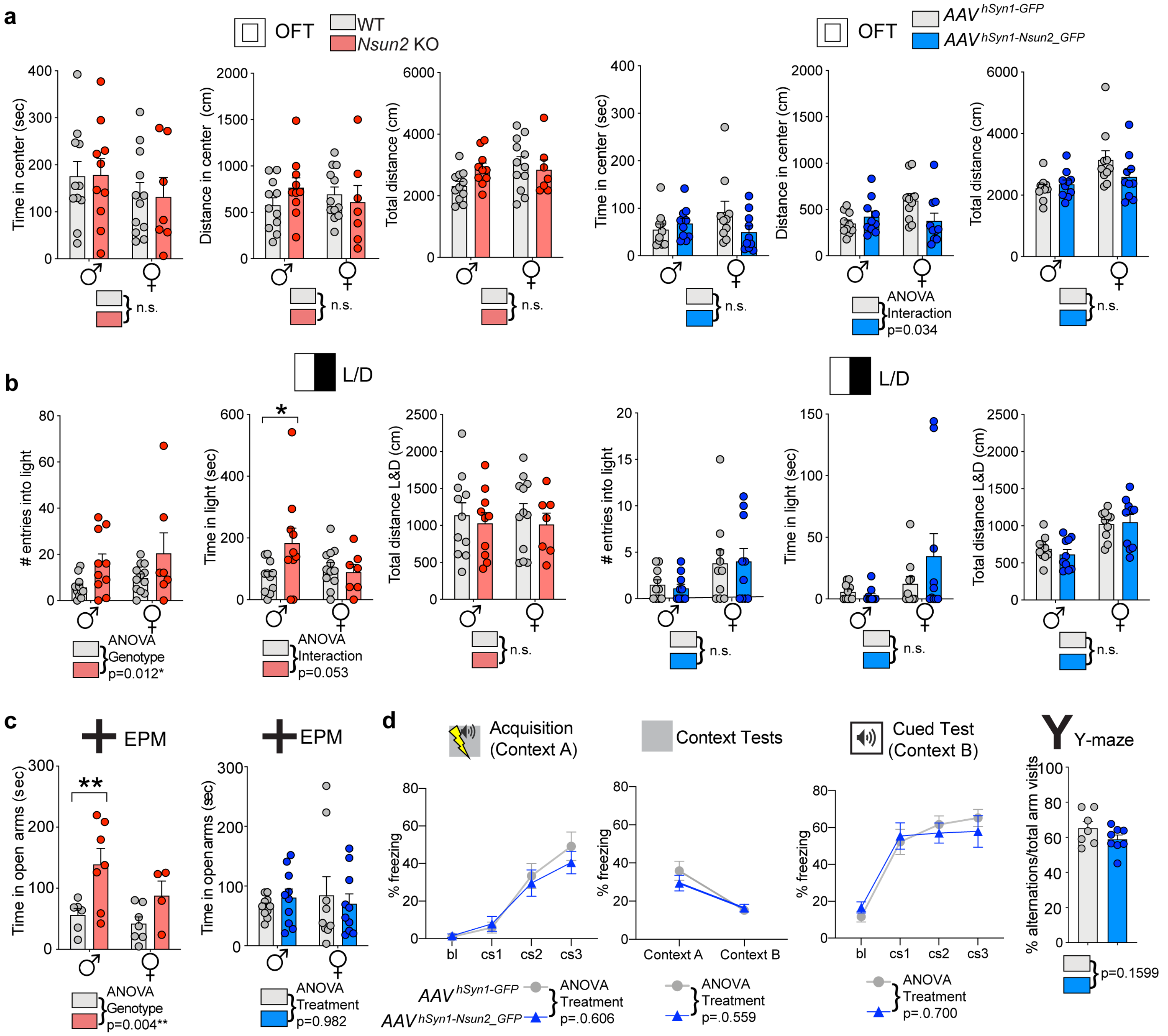

Supplementary Figure 6. (a) Left, two-way ANOVA revealed no significant differences in the OFT after Nsun2 ablation (n=11 WT M, 12 WT F, 10 KO M, 7 KO F). Right, an AAV injection x sex interaction was found for distance travelled in the center of the OFT, with no significant differences found in the other measures of the OFT (n=10/sex/AAV injection). **(b)** Left, in the L/D test after Nsun2 ablation, there was a significant interaction between genotype and sex, such that Nsun2 KO males spent more time in the light side of the L/D box (n=11 WT M, 12 WT F, 10 KO M, 7 KO F, two-tailed t-test with Bonferroni correction, *p=0.026). Right, no significance differences in L/D test measures were detected after Nsun2 overexpression (n=10/sex/AAV injection). **(c)** Left, in the EPM, Nsun2 KO mice likewise show an anxiolytic phenotype with more time spent in

the open arms compared to WT mice (n=6 WT M, 7 WT F, 7 KO M, 4 KO F, two-tailed t-test with Bonferroni correction, **p=0.010). Right, Nsun2 overexpressing mice show no significant changes in time spent in open arms in the EPM (n=10 AAV^{GFP} M, 9 AAV^{GFP} F, 10 AAV^{Nsun2} M, 10 AAV^{Nsun2} F). **(d)** Behavioral tests of cognition in adult Nsun2 overexpressing mice vs. GFP-injected controls show no significant differences between conditions for fear conditioning (n=8/AAV injection) or Y-maze (n=7 AAV^{GFP}, 8 AAV^{Nsun2}). Statistical results of all behavioral tests are shown in Supplementary Table 5. All post-hoc t-tests are two-tailed with Bonferroni correction. All data are presented as mean values +/- SEM. Source data are available as a Source Data file.

Supplementary Table 1. Brain and body weights for adult Nsun2 KO and WT mice

	Measure	Two-way ANOVA: Main effects	p value	Post-hoc t-tests (two tailed with Bonferroni correction)
Nsun2 KO*: CK–Cre*;*Nsun2*$^{2lox/2lox}$ mutant (KO) vs. *WT*				*KO vs. WT*
Body/Brain weights n=18 WT (8 M, 10F)/15 KO (10 M, 5 F)	Body weight (g)	Genotype: F (1, 29) = 34.67<br>Sex: F (1, 29) = 93.85<br>Interaction: F (1,29) =4.282	p<0.0001***<br>p<0.0001***<br>p=0.048*	Males: t(29)=6.078, p<0.0001***<br>Females: t(29)=2.526, p=0.104
	Brain weight (mg)	Genotype: F (1, 29) =177.8<br>Sex: F (1, 29) = 7.258<br>Interaction: F (1,29) = 6.326	p<0.0001***<br>p=0.012*<br>p=0.018*	Males: t(29)=8.264, p<0.0001***<br>Females: t(29)=10.48, p<0.0001***
	Brain/body ratio	Genotype: F (1, 29) = 5.421<br>Sex: F (1, 29) = 143.1<br>Interaction: F (1,29) = 11.13	p=0.027*<br>p<0.0001***<br>p=0.002**	Males: t(29)=0.770, p>0.999<br>Females: t(29)=3.747, p=0.005**

Supplementary Table 2. Site-specific statistical analysis of bisulfite tRNA sequencing using individual two-tailed t-tests with FDR adjusted p-values.

GlyGCC								
	CamK-Cre^{+}*,Nsun*$^{2lox/2lox}$ vs. WT Cortex; n=4 WT/4 KO				*AAV-Nsun2 vs. AAV-GFP* PFC tissue punch; n=5 GFP/ 5 Nsun2			
Cytosine	WT mean	KO mean	t-statistic (df=6)	adjusted p value	AAV-GFP mean	AAV-Nsun2 mean	t-statistic (df=8)	adjusted p value
26	0.4088	0.3707	0.239	0.641	0.37	0.3618	0.060	0.964
28	0.3566	0.3487	0.089	0.641	0.3431	0.3988	0.816	0.812
30	0.3924	0.401	0.169	0.641	0.4023	0.3994	0.098	0.964
31	0.2614	0.2617	0.003	0.641	0.2908	0.2879	0.059	0.964
34	0.407	0.3727	0.443	0.641	0.3773	0.3922	0.319	0.964
35	0.159	0.1822	0.638	0.641	0.1498	0.1314	0.674	0.824
37	85.84	84.2	2.993	0.034	86.23	87.12	2.412	0.235
39	20.87	8.654	11.670	***<0.0001	20.95	27.56	4.104	*0.038
46	93.91	45.97	59.140	***<0.0001	93.91	94.92	1.901	0.347
47	95.27	45.18	45.880	***<0.0001	95.11	96.11	1.422	0.429
48	92.4	43.07	57.430	***<0.0001	91.83	93.62	1.678	0.366

AspGTC								
	CamK-Cre^{+}*,Nsun*$^{2lox/2lox}$ vs. WT Cortex; n=4 WT/4 KO				*AAV-Nsun2 vs. AAV-GFP* PFC tissue punch; n=5 GFP/ 5 Nsun2			
Cytosine	WT mean	KO mean	t-statistic (df=6)	adjusted p value	AAV-GFP mean	AAV-Nsun2 mean	t-statistic (df=8)	adjusted p value
26	0.3478	0.3511	0.043	0.811	0.3556	0.4056	0.945	0.881
27	0.3031	0.3145	0.109	0.811	0.3138	0.339	0.407	0.881
28	0.3039	0.3415	0.391	0.811	0.3222	0.3523	0.451	0.881
29	0.3102	0.3332	0.359	0.811	0.3269	0.3509	0.505	0.881
31	0.3928	0.3938	0.024	0.811	0.4469	0.4931	0.837	0.881
32	0.2375	0.2509	0.191	0.811	0.2723	0.2507	0.607	0.881
36	0.4538	0.485	0.159	0.811	0.4608	0.4585	0.016	0.998
38	89.75	90.99	3.226	0.055	89.21	89.88	1.212	0.881
40	0.2909	0.3143	0.806	0.811	0.3224	0.3263	0.271	0.881
47	86.38	38.36	53.360	***<0.0001	87.28	88.82	1.722	0.881
48	93.3	41.07	46.160	***<0.0001	93.46	93.74	0.300	0.881

GluCTC								
	CamK-Cre^{+}*,Nsun*$^{2lox/2lox}$ vs. WT Cortex; n=4 WT/4 KO				*AAV-Nsun2 vs. AAV-GFP* PFC tissue punch; n=3 GFP/ 3 Nsun2			
Cytosine	WT mean	KO mean	t-statistic (df=6)	adjusted p value	AAV-GFP mean	AAV-Nsun2 mean	t-statistic (df=4)	adjusted p value
34	0.347	0.356	0.8175	0.560	0.3587	0.384	0.946	0.631
36	0.2496	0.1862	2.035	0.167	0.2119	0.2362	1.169	0.631
38	0.5004	0.4365	0.04079	0.340	0.4514	0.4493	0.030	0.987
39	0.4733	0.4993	0.3346	0.560	0.4386	0.5023	1.506	0.631
41	0.4667	0.5158	0.1234	0.537	0.4906	0.4457	0.940	0.631
42	0.5324	0.465	0.4031	0.340	0.4596	0.4784	0.325	0.855
44	0.4272	0.3599	21.06	0.167	0.3701	0.4137	0.753	0.631
47	13.42	5.582	19.95	***0.0001	13.3	18.37	1.490	0.631
48	91.6	49.76	144.9	***<0.0001	93.28	93.64	0.741	0.631
49	90.21	47.74	206.2	***<0.0001	91.45	92.42	1.790	0.631

GluTTC				
	CamK-Cre^{+}*,Nsun*$^{2lox/2lox}$ vs. WT Cortex; n=2 WT/2 KO		*AAV-Nsun2 vs. AAV-GFP* PFC punch; n=2 GFP/ 2 Nsun2	
Cytosine	WT mean	KO mean	AAV-GFP mean	AAV-Nsun2 mean
27	0.834	0.694	0.577	0.648
28	0.419	0.359	0.272	0.366
36	0.386	0.384	0.236	0.280
38	0.548	0.595	0.471	0.457
39	0.593	0.585	0.455	0.445
40	0.396	0.388	0.222	0.338
44	1.116	1.603	1.122	1.097
47	8.739	3.659	8.904	15.700
48	91.760	44.330	93.930	95.020
49	90.950	42.820	92.650	93.690

Supplementary Table 3. Downregulated proteins in KO vs. WT involved in excitatory synaptic signaling and neurotransmission and glycine content.

Gene/protein	T-test diff.	adj. p value	% glycine	References (PMID)
Bsn	-0.16985	0.00071	9.00558	PMID: 12628169 PMID: 29915867
Cacna1b	-0.20457	0.01875	6.65808	PMID: 31630675 PMID: 24523520
Cacng8	-0.19369	0.01063	14.65721	PMID: 16222232
Calb1	-0.76845	0.00047	6.13027	PMID: 28253930
Camk4	-0.41801	0.00090	5.11727	PMID: 28592691
Casp3	-0.35078	0.00015	6.13718	PMID: 25653368 PMID: 24478350
Cdk5	-0.06569	0.02654	5.47945	PMID: 28760951 PMID: 26088971
Cdkl5	-0.07745	0.03703	5.72917	PMID: 30288694 PMID: 29977282
Chl1	-0.35392	0.00188	6.86518	PMID: 31182634 PMID: 20711454
Ctnnb1	-0.20143	0.00740	7.17029	PMID: 26255771
Dab1	-0.22114	0.04765	8.75000	PMID: 26635527 PMID: 21325502
Dlg1	-0.20290	0.00312	7.91367	PMID: 30067285 PMID: 20133708
Dlg2	-0.22509	0.00046	7.54527	PMID: 30067114 PMID: 32164788
Dlg4	-0.09411	0.03806	7.87293	PMID: 31263190 PMID: 29155979
Dlgap2	-0.17812	0.00226	4.72144	PMID: 28870203
Dlgap3	-0.23690	0.00027	11.18012	PMID: 28870203
Efnb2	-0.25586	0.00498	8.03571	PMID: 28978486
Fabp7	-0.30991	0.00346	6.38298	PMID: 26296243
Flot1	-0.41884	0.00007	4.90654	PMID: 20669324
Flot2	-0.38551	0.00007	5.84112	PMID: 27993509
Gprin1	-0.31685	0.00162	9.44206	PMID: 31875540
Gria2	-0.17629	0.01540	8.26727	PMID: 31300657
Gria3	-0.32956	0.00113	7.43243	PMID: 28762944
Grik2	-0.26392	0.01679	6.49496	PMID: 8094892
Grin2b	-0.20993	0.00482	6.74764	PMID: 20890276
Grm3	-0.26917	0.00514	5.68828	PMID: 12213275 PMID: 29079293
Grm5	-0.29369	0.00226	7.48130	PMID: 27395786 PMID: 29079293
Grm8	-0.36429	0.00063	6.38767	PMID: 12213277
Homer2	-0.30060	0.00140	3.10734	PMID: 9808459
Il1rapl1	-0.20567	0.00842	5.89928	PMID: 23785489 PMID: 21926414
Mapk1	-0.20635	0.03122	4.46927	PMID: 14976517
Ncam2	-0.33695	0.01545	7.16846	PMID: 26611261
Nedd4l	-0.09643	0.00178	5.67729	PMID: 25505317 PMID: 26843640
Nptxr	-0.27804	0.04217	9.33063	PMID: 25754824
Nrcam	-0.11160	0.01056	6.52557	PMID: 25143608
Nrgn	-0.53200	0.00679	23.07692	PMID: 33032807
Pak1	-0.24897	0.00019	4.96324	PMID: 18644395 PMID: 15295021
Pick1	-0.19787	0.00059	7.54717	PMID: 28057533 PMID: 18184314
Ptk2b	-0.18344	0.00140	4.55016	PMID: 28555636 PMID: 20071509
Rab5a	-0.52625	0.00356	6.04651	PMID: 16141272
Rims1	-0.20333	0.00403	6.69856	PMID: 30559150
Rin1	-0.42248	0.00212	7.36434	PMID: 27852895 PMID: 19830836
Shank2	-0.14454	0.00194	6.77507	PMID: 28179641 PMID: 29572432
Shank3	-0.16221	0.00089	9.07514	PMID: 28179641 PMID: 21423165
Snap25	-0.16580	0.00696	6.79612	PMID: 19735702
Snca	-0.22250	0.00431	14.28571	PMID: 12388586
Sqstm1	-0.25487	0.00247	8.11518	PMID: 23511975
Syn1	-0.31718	0.00194	10.19830	PMID: 22539848
Syngap1	-0.17256	0.00737	8.13433	PMID: 12598599
Syngr3	-0.12693	0.03844	11.35371	PMID: 31090538 PMID: 29398363

Supplementary Table 4. Significantly altered proteins involved in glycine/serine biosynthesis pathways in KO vs. WT cortex.

Gene/protein	T-test diff.	adj. p value
Psat1	0.100028	0.092483
Psph	0.83494	0.00016
Phgdh	0.176175	0.034091
Shmt2	0.217788	0.019344
Srr	-0.35662	0.00208

Supplementary Table 5. Behavioral phenotyping in adult Nsun2 KO or WT mice, C57Bl/6 mice with PFC injection of AAV^{Nsun2} or AAV^{GFP}, and $Nsun2^{2lox/2lox}$ mice with AAV^{Cre} or AAV^{GFP} injection.

Behavioral test	Behavioral measure	Two-way ANOVA: Main effects	p value	Post-hoc t-tests (two-tailed with Bonferroni correction)
Nsun2 KO: *CamK–Cre*;*Nsun2* $^{2lox/2lox}$ mutant (KO) vs. *WT*				*KO vs. WT*
FST		Genotype: F (1, 34) = 7.578	p=0.009**	Males: t(34)=2.168, p=0.0745
n=21 WT (10 M, 11F)/	Time immobile	Sex: F (1, 34) = 0.883	p=0.354	Females: t(34)=1.745, p=0.180
17 KO (10 M, 7 F)		Interaction: F (1, 34) = 0.037	p=0.849	
TST		Genotype: F (1, 34) = 1.150	p=0.291	
n=21 WT (10 M, 11F)/	Time immobile	Sex: F (1, 34) = 0.179	p=0.675	n/a
17 KO (10 M, 7 F)		Interaction: F (1, 34) = 0.009	p=0.924	
EPM		Genotype: F (1, 20) = 10.66	p=0.004**	Males: t(20)=3.164, p=0.010**
n=13 WT (6 M, 7F)/	Time in open arms	Sex: F (1, 20) = 2.745	p=0.113	Females: t(20)=1.556, p=0.271
11 KO (7 M, 4 F)		Interaction: F (1, 20) = 0.878	p=0.360	
L/D		Genotype: F (1, 36) = 6.946	p=0.012*	Males: t(36)=1.910, p=0.128
n=23 WT (11 M, 12F)/	Entries into light	Sex: F (1, 36) = 1.130	p=0.295	Females: t(36)=1.824, p=0.153
17 KO (10 M, 7 F)		Interaction: F (1, 36) = 0.003	p=0.959	
		Genotype: F (1, 36) = 2.360	p=0.133	Males: t(36)=2.616, p=0.026*
	Time in light	Sex: F (1, 36) = 1.179	p=0.285	Females: t(36)=0.317, p>0.999
		Interaction: F (1, 36) = 4.011	p=0.053#	
		Genotype: F (1, 36) = 0.590	p=0.448	
	Total distance (L&D)	Sex: F (1, 36) = 0.000	p=0.994	n/a
		Interaction: F (1, 36) = 0.007	p=0.934	
OFT		Genotype: F (1, 36) = 0.000	p=0.981	
n=23 WT (11 M, 12F)/	Time in center	Sex: F (1, 36) = 1.698	p=0.201	n/a
17 KO (10 M, 7 F)		Interaction: F (1, 36) = 0.015	p=0.904	
		Genotype: F (1, 36) = 0.263	p=0.611	
	Distance in center	Sex: F (1, 36) = 0.030	p=0.863	n/a
		Interaction: F (1, 36) =1.672	p=0.204	
		Genotype: F (1, 36) = 0.769	p=0.386	
	Total distance	Sex: F (1, 36) = 2.218	p=0.145	n/a
		Interaction: F (1, 36) = 2.805	p=0.103	
Y maze n=7 WT, 7 KO	% spontaneous alternation	n/a	n/a	Sexes pooled t(12)=1.111, p=0.288
		Genotype: F(1,17)=3.929	p=0.064	cs1: t(51)=0.407, p>0.999
	Acquisition	CS: F(2,34)= 9.304	p=0.001***	cs2: t(51)=0.776, p>0.999
		Interaction: (2, 34) = 1.038	p=0.365	cs3: t(51)=2.311, p=0.075
Fear conditioning n=12 WT, 7 KO (2 way repeated measures ANOVA)		Genotype: F (1, 17) = 17.77	p=0.001***	Context A: t(34)=4.740, p<0.0001
	Contextual	Context: F (1, 17) = 64.43	p<0.0001***	Context B: t(34)=2.134, p=0.080
		Interaction: F (1, 17) = 5.064	p=0.038*	
		Genotype: F (1, 17) = 0.013	p=0.909	n/a
	Cued	CS: F (2,34) =12.87	p<0.0001***	
		Interaction: F (2,34) = 0.8365	p=0.442	

Nsun2 overexpression (OE): $AAV8^{hSyn1\text{-}Nsun2\_GFP}$ vs. $AAV8^{hSyn1\text{-}GFP}$				AAV^{Nsun2} vs. AAV^{GFP}
FST		AAV: $F(1, 32) = 8.947$	$p=0.005$**	Males: $t(32)=4.035$, $p=0.002$**
n=20 AAV^{GFP} (10 M, 10 F)	Time immobile	Sex: $F(1, 32) = 8.712$	$p=0.006$**	Females: $t(32)=0.090$, $p>0.999$
16 AAV^{Nsun2} (6 M,10F)/		Interaction: $(1, 32) = 9.669$	$p=0.004$**	
TST		AAV: $F(1, 36) = 10.270$	$p=0.003$**	Males: $t(36)=2.396$, $p=0.044$*
n=20 AAV^{GFP} (10 M,10F)/	Time immobile	Sex: $F(1, 36) = 0.305$	$p=0.584$	Females: $t(36)=2.136$, $p=0.079$
20 AAV^{Nsun2} (10 M, 10 F)		Interaction: $F(1, 36) = 0.034$	$p=0.855$	
EPM		AAV: $F(1, 35) = 0.001$	$p=0.982$	
n=19 AAV^{GFP} (10 M,9F)/	Time in open arms	Sex: $F(1, 35) = 0.050$	$p=0.824$	n/a
20 AAV^{Nsun2} (10 M, 10 F)		Interaction: $F(1, 35) = 0.609$	$p=0.440$	
L/D		AAV: $F(1, 36) = 0.009$	$p=0.926$	
n=20 AAV^{GFP} (10 M,10F)/	Entries into light	Sex: $F(1, 36) = 5.921$	$p=0.020$*	n/a
20 AAV^{Nsun2} (10 M, 10 F)		Interaction: $F(1, 36) = 0.079$	$p=0.781$	
		AAV: $F(1, 36) = 0.997$	$p=0.325$	
	Time in light	Sex: $F(1, 36) = 3.904$	$p=0.056$	n/a
		Interaction: $F(1, 36) = 1.723$	$p=0.198$	
		AAV: $F(1, 36) = 0.114$	$p=0.738$	
	Total distance (L&D)	Sex: $F(1, 36) = 26.530$	$p<0.0001$***	n/a
		Interaction: $F(1, 36) = 0.444$	$p=0.510$	
OFT		AAV: $F(1, 36) = 0.890$	$p=0.352$	
n=20 AAV^{GFP} (10 M,10F)/	Time in center	Sex: $F(1, 36) = 0.375$	$p=0.544$	n/a
20 AAV^{Nsun2} (10 M, 10 F)		Interaction: $F(1, 36) = 3.145$	$p=0.085$	
		AAV: $F(1, 36) = 1.184$	$p=0.284$	Males: $t(36)=0.793$, $p>0.999$
	Distance in center	Sex: $F(1, 36) = 2.318$	$p=0.137$	Females: $t(36)=2.332$, $p=0.153$
		Interaction: $F(1, 36) = 4.883$	$p=0.034$*	
		AAV: $F(1, 36) = 0.974$	$p=0.330$	
	Total distance	Sex: $F(1, 36) = 7.367$	$p=0.010$*	n/a
		Interaction: $F(1, 36) = 2.519$	$p=0.121$	
Y maze<br>n=7 AAV^{GFP}, 8 AAV^{Nsun2}	% spontaneous aternation	n/a	n/a	Sexes pooled<br>$t(13)=1.491$, $p=0.160$
		AAV: $F(1,14)=0.278$	$p=0.606$	
	Acquisition	CS: $F(2,28)= 38.78$	$p=0.0001$***	n/a
		Interaction: $(2, 28) = 0.739$	$p=0.487$	
Fear conditioning n=8 AAV^{GFP}, 8 AAV^{Nsun2} (2 way repeated measures ANOVA)		AAV: $F(1, 14) = 0.357$	$p=0.560$	
	Contextual	Context: $F(1, 14) = 45.39$	$p<0.0001$***	n/a
		Interaction: $F(1, 14) = 2.316$	$p=0.150$	
		AAV: $F(1, 14) = 0.155$	$p=0.700$	
	Cued	CS: $F(2,28) =1.560$	$p=0.228$	n/a
		Interaction: $F(2,28) = 0.7310$	$p=0.490$	

Nsun2 KO: $AAV8^{hSyn1\text{-}Cre\text{-}GFP}$ vs. $AAV8^{hSyn1\text{-}GFP}$				AAV^{Cre} vs. AAV^{GFP}
FST		AAV: $F(1, 30) = 2.439$	$p=0.129$	
n=16 AAV^{GFP} (7 M, 9F)/	Time immobile	Sex: $F(1, 30) = 6.464$	$p=0.016$**	n/a
18 AAV^{Cre} (8 M, 10F)		Interaction: $F(1, 30) = 1.648$	$p=0.209$	
TST		AAV: $F(1, 30) = 10.92$	$p=0.003$**	Males: $t(30)=0.446$, $p>0.999$
n=16 AAV^{GFP} (7 M, 9F)/	Time immobile	Sex: $F(1, 30) = 1.309$	$p=0.262$	Females: $t(30)=4.475$, $p=0.0002$***
18 AAV^{Cre} (8 M, 10F)		Interaction: $F(1, 30) = 6.957$	$p=0.013$**	

Supplementary Table 6. Site-specific statistical analysis of $tRNA^{Gly}{}_{GCC}$ bisulfite tRNA sequencing for Nsun2 2lox/2lox mice injected with AAV^{Cre} or AAV^{GFP} for PFC-specific Nsun2 KO (n=3/AAV injection; Individual two-tailed t-tests with FDR-adjusted p-values).

Cytosine	t-statistic (df=4)	adj. p value
26	1.123	0.291285
28	1.126	0.291285
30	0.6864	0.428368
31	1.167	0.291285
34	1.276	0.291285
35	0.3887	0.526904
37	2.02	0.183472
39	4.004	0.032486*
46	10.25	0.001728**
47	9.664	0.001728**
48	10.59	0.001728**

Supplementary Table 7. Primer sequences

**tRNA bisulfite sequencing mouse primer sequences for methylation analysis (without Illumina adapter sequence).**		
Isoacceptor	Forward Primer	Reverse primer
GlyGCC	5'-GGTGGTTTAGTGGTAGAATT-3'	5'-TACATAAACCAAAATC-3'
GluTTC	5'-TATATGGTTTAGTGG-3'	5'-TTCCCACACCAAAAATC-3'
GluCTC	5'-TCCCTGGTGGTCTAGTGG-3'	5'-TTCCCTAACCAAAAATC-3'
AspGTC	5'-TGTTAGTATAGTGGTGAGTAT-3'	5'-CTCCCCATCAAAAAATTA-3'
ValAAC	5'-GTTTTTGTAGTGTAGTGGTTAT-3'	5'-TATTTCCACCCAATTTCAAACC-3'
ProTGG	5'-GGTTTGTTGGTTTAG-3'	5'-AAACTCATCCAAAATTT-3'
**Full length tRNA Primer sets (Arraystar Inc.)**		
Gene/Isoacceptor	Cat. #	
GlyGCC	AS-NR-001M-1-064	
AspGTC	AS-NR-001M-1-033	
5s rRNA	AS-NR-001M-1-187	
**tRNA fragment custom Taqman probes**		
Gene/Isoacceptor	Target Sequence submitted	
GlyGCC 5'	GCAUUGGUGGUUCAGUGGUAGAAUUCUCGCC	
GluCTC 5'	UCCCUGGUGGUCUAGUGGUUAGGAUUCGGCGCUC	
GluTTC 5'	UCCCUGGUGGUCUAGUGGCUAGGAUUCGGCGCUU	
GluTTC full length	Full sequence	
5S rRNA full length	Full sequence	
**mRNA Taqman probes**		
Gene	Assay ID	
Nsun2	Mm01349532_m1	
Gapdh	Mm99999915_g1	
