# Supplementary material for: Neuronal Nsun2 deficiency produces tRNA epitranscriptomic alterations and proteomic shifts impacting synaptic signaling and behavior
Source: Nat Commun. 2021 Aug 13;12:4913. doi: 10.1038/s41467-021-24969-x (PMC8363735; doi:10.1038/s41467-021-24969-x)
Supplement: Supplementary file 3 — Reporting Summary [file 41467_2021_24969_MOESM3_ESM.pdf]

nature research

Corresponding author(s): Schahram Akbarian
Last updated by author(s): 7-3-21

Reporting Summary

Nature Research wishes to improve the reproducibility of the work that we publish. This form provides structure for consistency and transparency in reporting. For further information on Nature Research policies, see our Editorial Policies and the Editorial Policy Checklist.

Statistics

For all statistical analyses, confirm that the following items are present in the figure legend, table legend, main text, or Methods section.

n/a	Confirmed	
☐	☒	The exact sample size (*n*) for each experimental group/condition, given as a discrete number and unit of measurement
☐	☒	A statement on whether measurements were taken from distinct samples or whether the same sample was measured repeatedly
☐	☒	The statistical test(s) used AND whether they are one- or two-sided<br>*Only common tests should be described solely by name; describe more complex techniques in the Methods section.*
☐	☒	A description of all covariates tested
☐	☒	A description of any assumptions or corrections, such as tests of normality and adjustment for multiple comparisons
☐	☒	A full description of the statistical parameters including central tendency (e.g. means) or other basic estimates (e.g. regression coefficient) AND variation (e.g. standard deviation) or associated estimates of uncertainty (e.g. confidence intervals)
☐	☒	For null hypothesis testing, the test statistic (e.g. *F*, *t*, *r*) with confidence intervals, effect sizes, degrees of freedom and *P* value noted<br>*Give P values as exact values whenever suitable.*
☒	☐	For Bayesian analysis, information on the choice of priors and Markov chain Monte Carlo settings
☒	☐	For hierarchical and complex designs, identification of the appropriate level for tests and full reporting of outcomes
☐	☒	Estimates of effect sizes (e.g. Cohen's *d*, Pearson's *r*), indicating how they were calculated

Our web collection on statistics for biologists contains articles on many of the points above.

Software and code

Policy information about availability of computer code

Data collection	Commercially available sofware: For electrophysiological data collection, we used Digidata 1322A data acquisition system (Molecular Devices). For behavioral data collection, we used Fusion 5.0 Superflex system (Omnitech Electronics), Ethovision (v.14) software (Noldus), and Med Associates Video Freeze Software (Med Associates).
Data analysis	Commercially available software: For electrophysiological data analysis, we used pClamp Software (version 10.7; Molecular Devices) and Mini Analysis 6 (Synaptosoft). For proteomics analysis we used MaxQuant v. 1.6.6.0 and Perseus v. 1.6.5.0 software. For amino acid metabolomics analysis we used Skyline Daily v. 20.2.1.315. For statistical analyses and to create graphs, we used Graphpad Prism 8.4.3. For immunohistochemical image analyses and western blot analyses we used NIH ImageJ Software.<br>Open source: For RNAseq analyses, we usedSTAR (v2.5.3a) and featureCounts (subread v.1.5.2). For tRNA sequencing analyses we used FastQC v0.11.7 (available online at www.bioinformatics.babraham.ac.uk/projects/fastqc), cutadapt 1.17, BWA0.7.12-r1039, and edgeR. For bisulfite tRNA sequencing analysis we used the publicly available webserver bisAMP, available online at https://bisamp.dkfz.de/. For YAMAT/UMI seq we have provided open source code at https://github.com/goodarzilab/matRseq and for translation efficiency/codon content analysis we have provided open source code at https://github.com/goodarzilab/PAGE and https://github.com/goodarzilab/FIRE. For Riboseq data analysis we used cutadapt (v3.1), CLIPflexR v0.1.19, umi_tools v1.1.1, Bowtie2 v2.4.2, samtools v1.11, and umi_tools v1.1.1, with QC performed using Ribolog v0.0.0.9.

For manuscripts utilizing custom algorithms or software that are central to the research but not yet described in published literature, software must be made available to editors and reviewers. We strongly encourage code deposition in a community repository (e.g. GitHub). See the Nature Research guidelines for submitting code & software for further information.

Data

Policy information about availability of data

All manuscripts must include a data availability statement. This statement should provide the following information, where applicable:

- Accession codes, unique identifiers, or web links for publicly available datasets
- A list of figures that have associated raw data
- A description of any restrictions on data availability

Raw and processed mouse RNA sequencing data that support the findings of this study have been deposited the Gene Expression Omnibus (GEO) database under the accession number GSE165202. The mass spectrometry proteomics data have been deposited to the ProteomeXchange Consortium via the PRIDE68 partner repository with the dataset identifier PXD023437. Data supporting findings for this study are available within the Supplementary Information and Source Data file.. Related data are available from the corresponding author upon reasonable request. We report no restrictions on data availability.

Field-specific reporting

Please select the one below that is the best fit for your research. If you are not sure, read the appropriate sections before making your selection.

[x] Life sciences [ ] Behavioural & social sciences [ ] Ecological, evolutionary & environmental sciences

For a reference copy of the document with all sections, see nature.com/documents/nr-reporting-summary-flat.pdf

Life sciences study design

All studies must disclose on these points even when the disclosure is negative.

Sample size	No statistical tests for sample sizes were conducted. Sample sizes were chosen based on availability of animals after breeding.
Data exclusions	Two datapoints were excluded from the manuscript by preestablished standards of using Grubb's ESD outlier test. n=1 KO excluded from AspGTC tRNA qPCR analysis (Extended Data Figure 4a) and n=1 WT excluded from Nsun2 western blot analysis (Figure 1a).
Replication	We confirmed KO of Nsun2 via protein (western blot) and RNA (RNA seq, qPCR) assays and found consistent downregulation of Nsun2. We performed three independent analysis of tRNA expression levels to verify changes in Glycine tRNA expression (dm-HydrotRNAseq, YAMAT-seq, and qPCR) in the Nsun2 KO, and all methods independently supported the finding that Gly tRNA was depleted following NSun2 KO. We also confirmed tRNA fragment results by using qPCR and also confirmed a decrease in Gly tRNA fragments. All additional attempts at replication for individual experiments were successful.
Randomization	Allocation into experimental groups was randomly assigned.
Blinding	Experimenters were blind to condition during all behavioral testing and molecular assays until after data analysis steps when conditions were assigned.

Reporting for specific materials, systems and methods

We require information from authors about some types of materials, experimental systems and methods used in many studies. Here, indicate whether each material, system or method listed is relevant to your study. If you are not sure if a list item applies to your research, read the appropriate section before selecting a response.

Materials & experimental systems

n/a	Involved in the study	
[ ]	[x]	Antibodies
[x]	[ ]	Eukaryotic cell lines
[x]	[ ]	Palaeontology and archaeology
[ ]	[x]	Animals and other organisms
[x]	[ ]	Human research participants
[x]	[ ]	Clinical data
[x]	[ ]	Dual use research of concern

Methods

n/a	Involved in the study	
[x]	[ ]	ChIP-seq
[x]	[ ]	Flow cytometry
[x]	[ ]	MRI-based neuroimaging

Antibodies

Antibodies used	For western blot, we used Nsun2 polyclonal antibody (Proteintech, Cat #20854-1-AP), actin (Cell Signalling, Cat#4970S), histone H3 (Novus Biologicals, Cat#NB500-171), Synaptophysin (Abcam, Cat#ab8049), Neurogranin (Proteintech, Cat #10440-1-AP). For immunohistochemistry, we used NeuN antibody (Millipore Sigma, MAB377A5)
Validation	Full length western blots are presented in Extended Data Figure 1b and Extended Data Figure 5f and confirm expected sizes of

	protein bands from manufacturer's websites.

Animals and other organisms

Policy information about studies involving animals; ARRIVE guidelines recommended for reporting animal research

Laboratory animals	The study involved adult 3-6 month old C57Bl/6 male and female mice and 3-6 month old male and female conditional Nsun2 KO mice bred with a C57BL/6 background.
Wild animals	The study did not involve wild animals.
Field-collected samples	The study did not involve field-collected samples.
Ethics oversight	Institutional Animal Care and Use Committee of the Icahn School of Medicine at Mount Sinai

Note that full information on the approval of the study protocol must also be provided in the manuscript.
